# Reflection, reflective practice and reflective knowledge in nursing: where are we? A perspective

**DOI:** 10.1177/17449871251407833

**Published:** 2026-01-27

**Authors:** Sue Schutz

**Affiliations:** Senior Lecturer, Faculty of Health, Science and Technology, Oxford School of Nursing and Midwifery, Oxford Brookes University, Oxford, UK

**Keywords:** nurses, nursing knowledge, nursing practice, reflection, reflective practice

## Introduction

In our history, we can see that nurses bring together a range of influences and knowledge for creating solutions to practice dilemmas. Of the myriad literature published about reflective practice in nursing, the majority has been concerned with individual retrospective reflection around practice situations with the remainder addressing philosophical and theoretical aspects of reflection. Still, we lack a unified understanding of what we mean when we talk about reflection and reflective practice. So, what do we, as a community of practice, understand by the term reflection and how does it differ from reflective practice?

When we talk about reflection, we generally are referring to an activity that takes place after an event, what Schön (1983) named reflection-on-action, and others expanded to include reflection-in-action and reflection-before-action. Reflective practice is a term that has become somewhat synonymous with reflection but is more usefully referring to the use of reflection in the immediate context of daily professional life (Eraut, 1995) and a way of practising nursing that includes the use of reflection-on-action.

Perhaps some working definitions would help:

*Reflection* is a purposeful activity that is a retrospective process of reviewing practice in order to describe, analyse, and evaluate it for the purpose of learning about that practice.

*Reflective practice* is the use of a range of affective and cognitive abilities in the context of immediate professional practice, to address uncertainty and challenges in practice. It is a way of going about practice that is different from that traditionally established: by utilising personal knowledge gained from practice as well as technical-rational knowledge whilst going about one's work.

(Schutz, Unpublished Thesis 2015:13)

Whilst reflection on our practice looks inwards to our own knowledge, skills, and development, reflective practice looks out into the world and uses intuition and vision. Schön’s original argument was based on a rejection of the total reliance on what he called ‘technical-rational’ sources of knowledge, what we might call traditional scientific knowledge generated from quantitative or experimental research (the ‘hard high ground’ as Schön named it). Schön’s premise was that we should use these tried and tested ways of learning but we ignore what we learn in other ways at our peril. Schön brought the realisation that knowledge gained through reflection is diminished by the dominance of technical-rational ways of knowing. Reflection is, he argued, a major means of generating and passing on professional knowledge through practice itself.

The ‘situatedness’ of nursing means that we cannot rely on one source of evidence to inform practice if we are to practice person-centredness; when we are in the ‘swampy lowland’ of practice where traditional sources of evidence are not available or relevant, we have to:

. . . reconsider choices, sift through what one knows, and arrive at new ways of thinking and acting. (Sherwood, 2024: 401)

Carper (1978) proposed four fundamental ways of knowing in nursing that aimed to elucidate the many sources of knowledge that nurses use. These are: empirical knowing – the science of nursing, knowledge from research, theory, observation, and measurable evidence; ethical knowing – moral considerations, knowledge about values, obligations, and what ought to be done; personal knowing – self knowledge and the therapeutic use of self, knowledge gained through self-awareness and relationships with others; and aesthetic knowing – the art of nursing, nursings intuitive holistic understanding of a situation. Thorne (2020) described Carper’s work as part of nursing’s ‘disciplinary epistemological hardwiring’ which Carper described as intending to advance reflective practice and stem the *over-reliance* on pure science but not to reject it. Carper did not intend us to see these patterns as an either/or but emphasised their inter-relatedness. In viewing nursing as an art and a science, Carper’s work fosters reflective practice and emphasises the on-going process of knowing, being, and doing, rather than the static product of knowledge.

This on-going process of knowing, being, and doing is integral to reflective practice, where we constantly attempt to make sense of what we experience. This enables us to bring to our minds what we know instinctively (our tacit knowledge), and we see each new situation in the light of past experiences and knowing, learning afresh repeatedly:

In real-world practice, problems do not present themselves to the practitioner as givens. They must be constructed from the materials of problem situations which are puzzling, troubling, and uncertain.’ (Schön, 1983: 39–40)

Nursing is full of puzzling, troubling, and uncertain situations, and what we have seen and done in the past is what helps us to see and act in the present. Fundamentally, as Eraut (1995) argued, whilst our habitual practice *may* be sufficient, we cannot *assume* that it is. *Unthinking habitual practice* is what Socrates (in Plato’s Apology) refuted, saying ‘*the unexamined life is not worth living*’. It is critical self-reflection that gives our life value and meaning. Ultimately, what this leads us to is a practice where we think and act like researchers, as nurses with inquiring minds.

Thinking like a researcher is what defines the ‘true’ understanding of what reflection and reflective practice have to offer. When we think like a researcher in practice, we are looking for the clues that indicate whether our practice is effective or not: should we change the way we do something, find out more about the problem or re-think from the beginning? Through being reflective practitioners, we create a ‘spirit of inquiry’ (Sherwood, 2024: 401) and challenge our own assumptions (Atkins and Schutz, 2013) thereby seeing that being a reflective practitioner is not just about *doing* something, it is about *being* something. The something that we are being is reflexive.

Reflexivity, as defined by Finlay and Gough (2003) requires us to acknowledge our own role in knowledge generation, where our way of being meets how we generate new knowledge. Finlay describes reflexivity on her website (https://www.lindafinlay.co.uk/reflexivity/) as ‘*methodical, self-aware reflection*’. It is not an isolated event but a constant way of being. Philosophically this is the meeting point of *ontology* (the nature of our world) with *epistemology* (how new knowledge is created), and praxis (our way of being in the world). This more conceptual way of thinking about reflective practice brings us back in time. Not only did Socrates bid us to be reflective but also the later philosopher Aristotle conceived the notion of *Practical Wisdom* or *Phronesis*. Aristotle saw Phronesis as one form of knowledge that is part of his *Nicomachean Ethics*:

- Episteme – scientific and theoretical; generated through research; deductive, objective and reproducible
- Techne – art or craft; inductive; gained by personal experience (experiential) through creating or doing
- Phronesis – practical wisdom or knowledge gained through interaction and ethical understanding

Aristotle emphasised the importance of reflecting in the real world and developing experience of it, using our emotions, not in a way that gets in the way of ‘good’ rational thinking, but rather as a deliberative part of our thinking (Bulman, 2013). Jenkins et al. (2019) argued that it is the morally informed actions of the nurse that distinguishes the Aristotelian notion of phronesis; the nurse with practical wisdom makes decisions that are intellectually and ethically sound. But the focus is always on practice; like Schön in the 1980s, Ryle (1949) four decades earlier argued that having knowledge about something (knowing that) is not equal to knowledge of how (knowing how) to do something and knowing how precedes knowing that. Thus, Schön’s theories of the Reflective Practitioner, following the words of Aristotle, emphasised the need to learn from real life.

## What does reflective practice look like?

Atkins and Schutz (2013) used the work of Schön (1983, 1987) to conceptualise the reflective practitioner in nursing from multiple perspectives:

### A reflective practitioner demonstrates an artistic practice

An artistic practitioner uses a repertoire of past experiences to consider the best way to act in a situation. According to Schön (1987), this attribute reveals the knowledge that we hold in our ‘intelligent action’ and thus shows our ‘knowing in action’ (Schön, 1987).

### A reflective practitioner frames problems and experiments in practice

A reflective practitioner acts in a new situation as they have in others but uses a problem-framing approach rather than a problem-solving approach. An experienced reflective practitioner follows rules that have not yet been made explicit and invents new rules ‘on the spot’ (Schön, 1987) and behaves more like a researcher than an expert (Schön, 1987). This is what we might see in our practice when we try out new ways of approaching patient care problems that have not been amenable to previous practice interventions.

### A reflective practitioner has a transactional and constructivist relationship with practice

Here is the skill of awaiting the outcomes of the ‘on the spot’ experiment in practice. The reflective practitioner will observe the cues that guide them to discard or adopt a new response in practice. These cues might be found in the patient or client’s physical, mental, emotional, or spiritual well-being as defined by the individual.

### A reflective practitioner possesses tacit knowledge

We are all familiar with that instinct to behave in a certain way with patients and clients, to act or not to act, and to be able to discriminate between courses of action. Polanyi (1958) termed the knowledge that informs this *tacit knowledge*, the ‘we know more than we can tell’ mantra. Whilst being invaluable in our profession, we cannot use this form of knowledge unless we can bring it to our minds with clarity and form the words to articulate it through reflection.

### A reflective practitioner articulates their reflective practice

Learning from our reflective practice is, of course, valuable to us as individuals but we need to articulate what we have learned for the benefiit of the whole community. Reflections on practice enable us to bring to the surface our new and our tacit knowledge so that we can put this into words promoting both our scholarship and our knowledge development and that of others. Reflection plays an important part in how nursing knowledge is formed and communicated.

If being reflective is thinking like a researcher, then the epistemology of reflective practice – the knowledge generated – is often not valued (Mantzoukas and Jasper, 2004). The exception to this may be in nursing education, but the premise may also apply there too (Schutz, 2013). To ignore the value of reflective knowledge is to submit nursing to an epistemic injustice (Fricker, 2007). Promoting the importance of reflection is acknowledging the primacy of practice as our key way of coming to understand and articulate the value of nursing knowledge. A strong and sustained focus on the centrality of experiential knowledge, revealed through reflection, can transform nursing as a practice-based discipline. Nurses’ work, grounded in reflection, helps find meaning and purpose in the hard work of nursing, fosters joy, unearths satisfaction and teamwork, creating a cycle of renewal, energy, and a regenerative spirit. Understanding the nuances of reflection, reflective practice, and reflexivity can help educators and clinicians in guiding a spirit of inquiry as the foundation of knowing, being and doing.

## References

[bibr1-17449871251407833] Atkins S Schutz S (2013) Developing skills for reflective practice. Chapter 2, In: Bulman C Schutz S (eds) Reflective Practice in Nursing. Oxford: Wiley Blackwell.

[bibr2-17449871251407833] Bulman C (2013) An introduction to reflection. Chapter 1, In: Bulman C Schutz S (eds) Reflective Practice in Nursing. Oxford: Wiley Blackwell.

[bibr3-17449871251407833] Carper B (1978) Fundamental patterns of knowing in nursing. Advances in Nursing Science 1: 13–23. 110216 10.1097/00012272-197810000-00004

[bibr4-17449871251407833] Eraut M (1995) Schon Shock: A case for refraining reflection-in-action? Teachers and Teaching 1: 9–22.

[bibr5-17449871251407833] Finlay L Gough B (eds) (2003) Reflexivity: A Practical Guide for Researchers in Health and Social Sciences. Oxford: Blackwell Science Ltd.

[bibr6-17449871251407833] Fricker M (2007) Epistemic Injustice: Power and the Ethics of Knowing. Oxford: Oxford Academic. DOI: 10.1093/acprof:oso/9780198237907.001.0001.

[bibr7-17449871251407833] Jenkins K Kinsella EA Deluca S (2019) Perspectives on phronesis in professional nursing practice. Nursing Philosophy 20: e12231. DOI: 10.1111/nup.12231. 30450682

[bibr8-17449871251407833] Mantzoukas S Jasper MA (2004) Reflective practice and daily ward reality: a covert power game. Journal of Clinical Nursing 13(8): 925–33. 10.1111/j.1365-2702.2004.01008.x 15533098

[bibr9-17449871251407833] Polanyi M (1958) Personal Knowledge: Towards a Post-Critical Philosophy. London: Routledge & Kegan Paul.

[bibr10-17449871251407833] Ryle G (1949) The Concept of Mind. New York: Barnes and Noble.

[bibr11-17449871251407833] Schön DA (1983) The Reflective Practitioner: How Professionals Think in Action. New York: Basic Books.

[bibr12-17449871251407833] Schön DA (1987) Educating the Reflective Practitioner. San Francisco: Jossey Bass.

[bibr13-17449871251407833] Schutz S (2013) Assessing and evaluating reflection, Chapter 8. In: Bulman C Schutz S (Eds) Reflective Practice in Nursing.Oxford: Wiley Blackwell.

[bibr14-17449871251407833] Sherwood G (2024) Reflective practice and nursing knowledge development: rethinking evidence for a practice-based discipline. International Journal of Nursing Sciences. Epub ahead of print 8 August 2024. DOI: 10.1016/j.ijnss.2024.08.002 PMC11740302 39830915

[bibr15-17449871251407833] Thorne S (2020) Rethinking Carper’s personal knowing for 21st century nursing. Nursing Philosophy 21: e12307. DOI: 10.1111/nup.12307. PMC7583479 32567117

